# Artificial Intelligence in Dental Caries Diagnosis and Detection: An Umbrella Review

**DOI:** 10.1002/cre2.70004

**Published:** 2024-08-29

**Authors:** Sapna Negi, Ankita Mathur, Snehasish Tripathy, Vini Mehta, Niher Tabassum Snigdha, Abdul Habeeb Adil, Mohmed Isaqali Karobari

**Affiliations:** ^1^ Department of Dental Research Cell, Dr. D. Y. Patil Dental College and Hospital Dr. D. Y. Patil Vidyapeeth Pune Maharashtra India; ^2^ Department of Dental Research, Saveetha Medical College and Hospitals, Saveetha Institute of Medical and Technical Sciences Saveetha University Chennai Tamil Nadu India; ^3^ Department of Restorative Dentistry & Endodontics, Faculty of Dentistry University of Puthisastra Phnom Penh Cambodia

**Keywords:** artificial intelligence, dental caries, neural networks, umbrella review

## Abstract

**Background and Aim:**

Dental caries is largely preventable, yet an important global health issue. Numerous systematic reviews have summarized the efficacy of artificial intelligence (AI) models for the diagnosis and detection of dental caries. Therefore, this umbrella review aimed to synthesize the results of systematic reviews on the application and effectiveness of AI models in diagnosing and detecting dental caries.

**Methods:**

MEDLINE/PubMed, IEEE Explore, Embase, and Cochrane Database of Systematic Reviews were searched to retrieve studies. Two authors independently screened the articles based on eligibility criteria and then, appraised the included articles. The findings are summarized in tabulation form and discussed using the narrative method.

**Result:**

A total of 1249 entries were identified out of which 7 were finally included. The most often employed AI algorithms were the multilayer perceptron, support vector machine (SVM), and neural networks. The algorithms were built to perform the segmentation, classification, caries detection, diagnosis, and caries prediction from several sources, including periapical radiographs, panoramic radiographs, smartphone images, bitewing radiographs, near‐infrared light transillumination images, and so forth. Convoluted neural networks (CNN) demonstrated high sensitivity, specificity, and area under the curve in the caries detection, segmentation, and classification tests. Notably, AI in conjunction with periapical and panoramic radiography images yielded better accuracy in detecting and diagnosing dental caries.

**Conclusion:**

AI models, especially convolutional neural network (CNN)‐based models, have an enormous amount of potential for accurate, objective dental caries diagnosis and detection. However, ethical considerations and cautious adoption remain critical to its successful integration into routine practice.

## 1 Introduction

Dental caries, often known as tooth decay, is a condition characterized by the demineralization of tooth structures caused by sugar‐driven cariogenic plaque bacteria (Kazeminia et al. 2020). Dental caries are largely preventable, yet an important global health issue affecting nearly two billion adults and 514 million children worldwide (WHO 2022). This burden is pronounced in Southeast Asian and Western Pacific nations, affecting disproportionately impoverished and socially disadvantaged populations (Jain et al. 2024). From an economic standpoint, the cost of dental caries involving both permanent and deciduous teeth reaches nearly US$ 22 billion and US$ 1.55 billion, respectively, putting a large indirect cost burden on society. In addition, the issue is compounded by the uneven distribution of dental professionals, with only 1.4% working in low‐income countries (Jain et al. 2024).

Early caries detection has the potential to not only prevent invasive treatments, such as selective or stepwise caries removal, and restoration treatments but also reduce the time and cost of treatment and alleviate the strain on the healthcare system (Moharrami et al. 2024). The standard diagnostic strategy used for caries detection is visual‐tactile inspection, in which early lesions, such as proximal, occlusal, pit, and fissure dental caries, are difficult to detect (Gimenez et al. 2015). Dental radiography, such as intraoral periapical radiography (IOPAs) and radiovisiography (RVGs), are common approaches in a typical clinical setting that provide a visual portrayal of the extent of the carious lesion (Muñoz‐Sandoval et al. 2022). Although dental radiography is more sensitive in detecting early lesions, it has a significant proportion of false‐positive or false‐negative detections because it is subjective and reliant on the examiner's experience (Schwendicke et al. 2021).

Digital health technologies and tools present excellent prospects for improving the diagnosis of oral problems. One such technology, known as artificial intelligence (AI), has recently become a research hotspot and an emerging trend in clinical care (Tripathy, Mathur, and Mehta 2023), with multiple studies indicating its potential in the field of dentistry (Ahmed et al. 2023; Mertens et al. 2021; Schwendicke et al. 2021). AI is a branch of computer science that displays the traits of human behavior (Patil et al. 2022). Numerous studies have examined the efficacy of AI models, including convolutional neural networks (CNNs) and artificial neural network algorithms, for the diagnosis, classification, segmentation, and prediction of dental caries (Mertens et al. 2021; Ramos‐Gomez et al. 2021; Zhu et al. 2022). The evidence from these investigations is summarized in a number of systematic reviews (Ahmed et al. 2023; Khanagar et al. 2022; Moharrami et al. 2024; Prados‐Privado et al. 2020).

Although studies have shown that AI is beneficial, its use has not yet been integrated into standard dental care, and the field is still in its infancy(Patil et al. 2022). Therefore, an umbrella review is crucial to obtain more precise and thorough findings on a certain subject. The findings of this study will inform dental practitioners about the practical application of AI, enable researchers to identify gaps in knowledge, and educate future dentists about evolving concepts and practices in the dental field. Furthermore, it will assist healthcare policymakers in developing policies for responsible AI integration, ultimately leading to enhanced patient care and more efficient dental caries diagnosis. Therefore, the goal of this extensive research is to summarize the findings of systematic reviews regarding the application and effectiveness of AI models in diagnosing and detecting dental caries.

## 2 Methodology

An umbrella review was carried out to summarize the findings of systematic reviews on the application and effectiveness of AI models in diagnosing and detecting dental caries using the following PICO elements:

P (Population): Patient dental image data sets.

I (Intervention): AI‐based models or algorithms for dental caries diagnosis and detection.

C (Comparator): Conventional methods/other algorithms/no comparator.

O (Outcome): Outcome metrics such as accuracy, sensitivity, specificity, area under curve (AUC), and so forth.

We followed the JBI's (Joanna Briggs Institute) umbrella review guidelines (JBI 2020) and PRISMA guidelines for reporting our study (Page et al. 2021). This review's protocol has been registered with PROSPERO under the registration number: CRD42023464376 (PROSPERO International Prospective Register of Systematic Reviews 2023).

### 2.1 Search Strategy

Two authors (S.N. and A.M.) searched the MEDLINE/PubMed, IEEE Explore, Embase, and Cochrane Database of Systematic Reviews bibliographic databases on August 18, 2023. These databases were chosen based on their comprehensive coverage across biomedical, technological, and evidence‐based peer‐reviewed literature. To find gray literature, we also used “Google Scholar” as a search engine. As Google Scholar retrieved many results, we only examined the first 100 results based on relevance. We also performed backward and forward reference list screening, or, more specifically, reviewed the reference lists and the citation list of included reviews, to find other studies that are pertinent to the review.

To build the search strategy, systematic reviews (SRs) that were relevant to the review were referred. The terms were selected in accordance with the target study design (a systematic review), target population (dental caries), and target intervention (AI‐based techniques). The thorough search stipulation utilized for searching each database is shown in Supporting Information S1: Table S1.

### 2.2 Study Eligibility Criteria

This review covered systematic reviews with or without meta‐analysis that concentrated on the application and effectiveness of AI‐based methods in managing dental caries without restrictions for data type (such as radiographic data, data, or clinical data), time of publication, setting, or language. Reviews that failed to demonstrate at least one of the accuracy, sensitivity, specificity, or area under the curve (AUC) measurements of classifier performance were disregarded. Scoping reviews, literature reviews, rapid reviews, criteria reviews, and other types of reviews lacking sound and consistent methodologies and critical appraisal techniques, as well as reviews where the main source of information is not original work, were also rejected. Additionally, we omitted editorials, preprints, commentaries, conference abstracts, or posters.

### 2.3 Study Selection, Data Extraction, and Quality Appraisal

The selection process was divided into two stages: first, two reviewers (S.N. and A.M.) independently examined the titles and abstracts of all papers that were retrieved; and second, the two reviewers read individually the complete texts of the papers that were selected in the previous step. To extract the data carefully and methodically, we created a form pilot‐tested initially on two studies. Two reviewers (S.N. and A.M.) separately retrieved data from the included reviews in a spreadsheet.

The quality of the included reviews was further evaluated separately by two reviewers (S.N. and A.M.) using the JBI Critical Appraisal Checklist for Systematic Reviews and Research Syntheses (JBI n.d.). For any discrepancies among the reviewers in all phases, settlement was performed through discussion with senior authors who are subject‐matter experts.

### 2.4 Data Synthesis

The data from primary investigations were summarized in many SRs, which did not offer definitive results. Additionally, the included reviews included a heterogeneity of data set types, AI classifiers, and metrics for classifier performance. We therefore used a two‐pronged strategy to address this variability. Thus, we presented the range of classifier measurements of performance findings. First, we tabulated the extracted data to assemble and organize the findings from the primary research as described in the SRs. Second, we used the vote‐counting method to conduct a synthesis of the retrieved data, counting and aggregating the reported findings from the included SRs to determine the overall trajectory of the evidence. Last, we used a narrative method to describe and evaluate the outcome of the available evidence. Thus, we presented the range of classifier measurements of performance findings.

## 3 Results

### 3.1 Search Results

**Figure 1:**
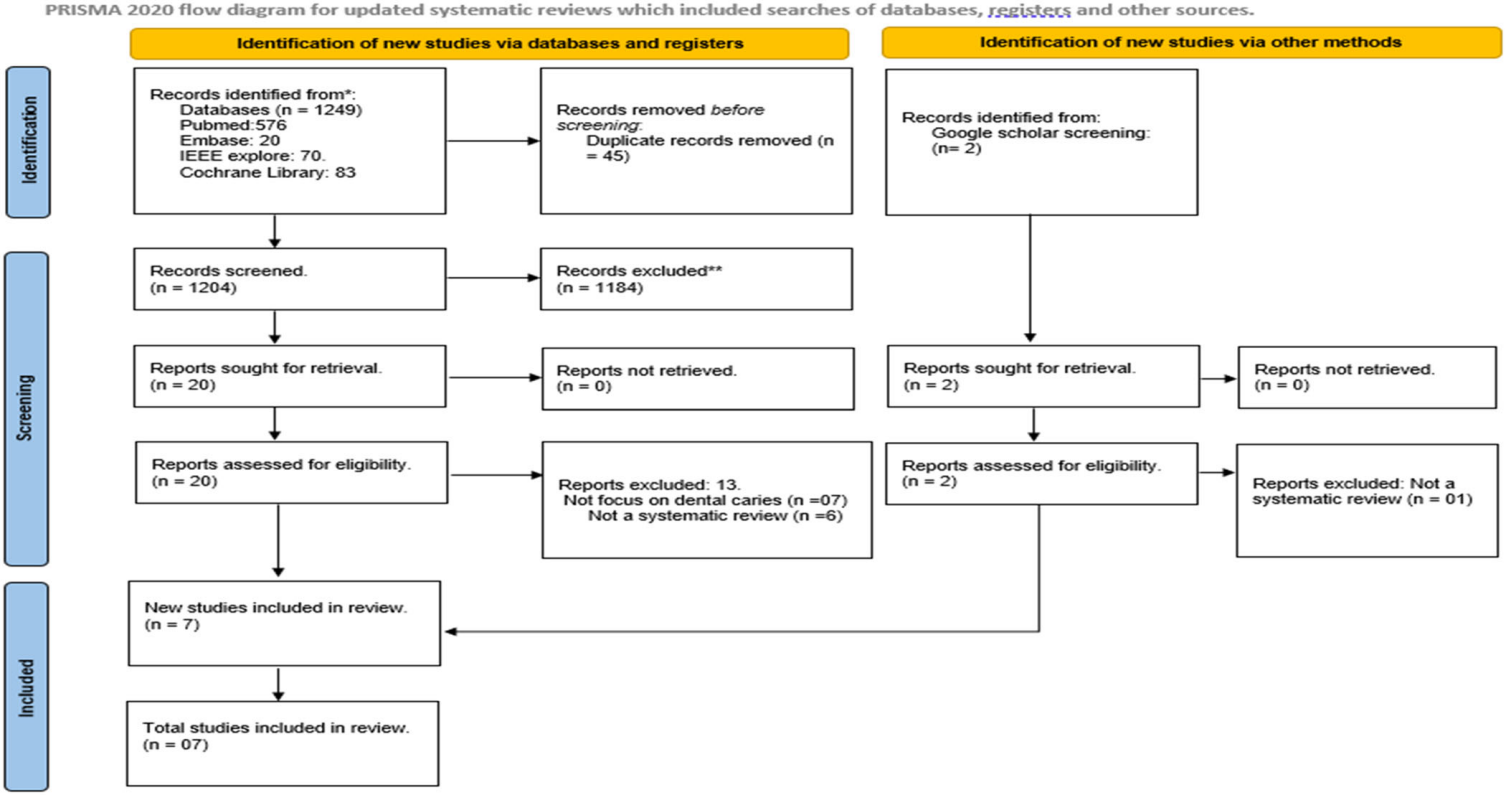
PRISMA flow diagram.

As illustrated in Figure 1 (PRISMA diagram), our search of the literature databases retrieved a total of 1249 entries. Rayyan software was used to identify and delete 45 duplicates from the citations. After reviewing the titles and abstracts of the remaining 1204 entries, 446 papers were further eliminated. The remaining 20 studies underwent full text analysis, but 13 of them were excluded because they either were not systematic reviews, or they failed to present data on dental caries. Thus, only 7 studies that met the objective and eligibility criteria of this umbrella review were included for further analysis (Khanagar et al. 2022; Mohammad‐Rahimi et al. 2022; Moharrami et al. 2024; Prados‐Privado et al. 2020; Revilla‐León et al. 2022; Reyes et al. 2022; Talpur et al. 2022).

### 3.2 Characteristics of Included Reviews

The systematic reviews included in this study were published between 2020 and 2023, with the majority (*n* = 5) publishing in 2022, as shown in (Table 1). Five of these reviews were conducted by reviewers from various nations, including Canada, Pakistan, Brazil, Saudi Arabia, and Spain. Notably, two reviews, by Mohammad‐Rahimi et al. (2022) and Revilla‐León et al. (2022) were carried out by a team of researchers from multiple nations. Surprisingly, only three of the studies (Mohammad‐Rahimi et al. 2022; Moharrami et al. 2024; Reyes et al. 2022) reported having a registered protocol in the PROSPERO registry. Four studies explicitly stated that they followed the Preferred Reporting Items for Systematic Reviews and Meta‐Analyses (PRISMA) guidelines. On the other hand, two studies (Khanagar et al. 2022; Mohammad‐Rahimi et al. 2022) indicated compliance with PRISMA‐DTA (Diagnostic Test Accuracy) guidelines, while one study (Prados‐Privado et al. 2020) did not state that the reporting guidelines were followed. In terms of study design, all the reviews included original studies, while two studies mentioned including conference proceedings as well (Moharrami et al. 2024; Prados‐Privado et al. 2020). Three reviews limited their inclusion criteria to papers that had been published in English (Khanagar et al. 2022; Moharrami et al. 2024; Talpur et al. 2022), whereas the other three applied no language constraints (Mohammad‐Rahimi et al. 2022; Prados‐Privado et al. 2020; Reyes et al. 2022). One study, nevertheless, failed to clarify if it had linguistic limits (Revilla‐León et al. 2022). There was a noticeable variation in the time range applied with three reviews considering research from inception (Moharrami et al. 2024; Prados‐Privado et al. 2020; Reyes et al. 2022), while three restricted their coverage to papers published within the last 10–12 years (Khanagar et al. 2022; Mohammad‐Rahimi et al. 2022; Talpur et al. 2022) and one study did not mention the timeline (Revilla‐León et al. 2022).

**Table 1:** Meta‐data of studies.

Author (year)	Year	Country	Protocol registration	Guidelines followed	Study designs included	Language restriction	Time limit
Prados‐Privado et al. (2020)	2020	Spain	NR	Not mentioned	Full manuscripts, including conference proceedings	None	Until 15 August 2020
Khanagar et al. (2022)	2022	Saudi Arabia	NR	PRISMA‐DTA	Prospective cohort, Retrospective Cohort, Cross sectional, RCT	English	January 2000 until February 2022
Revilla‐León et al. (2022)	2022	Spain, Texas, New York, Switzerland, and Boston	NR	PRISMA	Quasi‐experimental	NR	NR
Mohammad‐Rahimi et al. (2022)	2022	Germany, Iran, Latvia, and Chile	PROSPERO	PRISMA‐DTA	Original studies	none	2010–April 12, 2021
Talpur et al. (2022)	2022	Pakistan	NR	PRISMA	Original studies	English	2008–2022
Reyes et al. (2022)	2022	Brazil	PROSPERO	PRISMA	Longitudinal and cross‐sectional studies	None	Until December 28, 2020.
Moharrami et al. (2024)	2023	Canada	PROSPERO	PRISMA	Original article and conference proceedings	English	Upto December 15, 2022

The PICO format of all included studies is shown in Table 2. There was some variability across the PICO formats in studies. In terms of population, four studies considered patient radiographic image databases (Mohammad‐Rahimi et al. 2022, 2022; Reyes et al. 2022; Talpur et al. 2022). The intervention included AI applications (*n* = 3), neural networks (*n* = 2), and machine learning models (*n* = 2). The majority of studies have used expert judgment or clinical examination/reference tests or no comparator as a comparator group. Only Talpur et al. (2022) used different machine learning techniques for the prediction of caries as a comparator group. The outcome variables reported were varied across the studies with accuracy being the most common.

**Table 2:** PICO format of included studies.

Author (year)	Year	Population	Intervention	Comparator	Outcome
Prados‐Privado et al. (2020)	2020	Neural networks and caries detection	Caries definition and which teeth are detected	Studies with neural networks are used to detect and diagnose dental caries	Outcome metrics and value
Khanagar et al. (2022)	2022	Patients who underwent investigation for DC	AI applications for the detection, diagnosis, and prediction of DC	Expert/Specialist opinions, Reference standards/models	Accuracy, sensitivity, specificity, ROC = receiver operating characteristic curve, AUC = area under the curve, AUROC = area under the receiver operating characteristic, ICC = intraclass correlation coefficient, IOU = intersection‐over‐union, PRC = precision‐recall curve, statistical significance, F1 scores, vDSC = volumetric dice similarity coefficient, sDSC = surface dice similarity coefficient, PPV = positive predictive value, NPV = negative predictive value, mean decreased gini (MDG), mean decreased accuracy (MDA) coefficients, intersection over union (IoU), dice coefficient
Mohammad‐Rahimi et al. (2022)	2022	Studies used deep learning models on dental imagery resulting from routine care, clinical studies, or studies on extracted teeth	Caries classification, detection, or segmentation models with deep learning (NN) architecture	Reference test	Any kind of accuracy estimate on the image, tooth, surface, or pixel level
Revilla‐León et al. (2022)	2022	Clinical applications in restorative dentistry for the diagnosis of dental caries and vertical tooth fracture, detection of the tooth preparation finishing line, and prediction of restoration failure	Artificial intelligence learning	Not applicable	Diagnostic performance of the AI model for the diagnosis of dental caries and tooth fracture, the accuracy of tooth preparation finishing line location, and the prediction of restoration failure
Reyes et al. (2022)	2022	Data set obtained from human subjects (radiographic, photographic, or near‐infrared light transillumination [NILT] images, and medical records)	Diagnostic or prognostic prediction of dental caries assisted by non‐logistic regression (non‐LR) ML algorithms.	Expert judgment, clinical/histological examination, and classifiers reference as logistic regression (LR).	Analysis of ML performance in detection, diagnosis, or prognostic prediction of dental caries (outcomes such as accuracy/precision, sensitivity/recall, specificity, receiver operating characteristic curve, area under the curve, or positive/negative predictive values)
Talpur et al. (2022)	2022	Dental X‐ray images of human beings	Analysis of the different algorithms of machine learning used to detect dental caries.	Different algorithms of machine learning to predict the caries.	Accuracy of algorithms
Moharrami et al. (2024)	2023	Digital intraoral photographs from the oral cavity and teeth	Application of AI‐based models (DL and non‐DL) for caries detection	Reference standards such as clinical dental examinations or image annotations by dental professionals.	Performance metrics such as sensitivity, specificity, precision, F1‐score, accuracy, and area under receiver operating characteristics (AUROC).

### 3.3 Study Search, Appraisal, and Synthesis Methods in Included SRs

Varied numbers of electronic databases were searched in the included reviews with MEDLINE/PubMed (*n* = 7), EMBASE (*n* = 5), and Scopus (*n* = 5) being the most common. Out of seven, five studies reported reference list checking to identify further studies. The included SRs were critically assessed for methodological quality by all reviews using six different tools, such as a Cochrane risk of bias assessment tool (*n* = 1), JBI Critical Appraisal Checklist for Quasi‐Experimental Studies (*n* = 1), and a revised tool for Quality Assessment of Diagnostic Accuracy Studies (QUADAS‐2) (*n* = 4). Talpur et al. (2022) conducted the quality assessment, however, did not mention the name of the standard tool used. All reviews synthesized the data using a narrative approach due to wide heterogeneity across the AI model/algorithms used in the primary studies. Only Prados‐Privado et al. (2020) used descriptive measures, such as mean, standard deviation (SD), median, and percentage for data synthesis (Supporting Information S1: Table S2).

### 3.4 Search Results and Data Set Features in Included SRs

**Figure 2:**
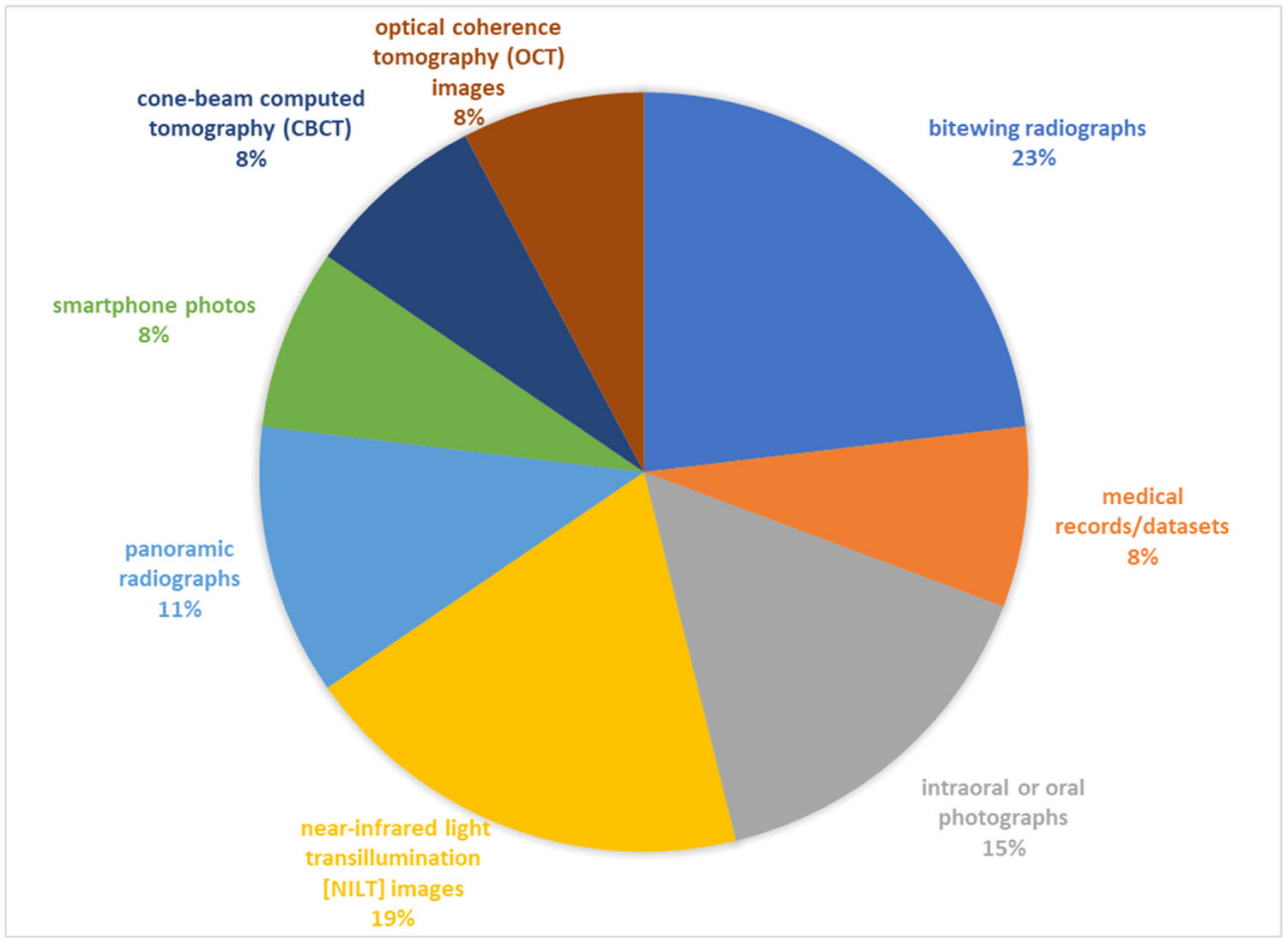
Variety of dental images used across studies.

The range of retrieved studies in the included reviews was 133–3410, whereas the range of included studies was 12–42. In the included studies, the size of the data sets utilized for training and validating the algorithms varied from 32 to 12,600 data sets (Supporting Information S1: Table S3). The included studies used a variety of dental images for training and validating the models, including periapical radiographs (*n* = 4), bitewing radiographs (*n* = 6), near‐infrared light transillumination (NILT) images (*n* = 5), intraoral or oral photographs (*n* = 4), panoramic radiographs (*n* = 3), smartphone photos (*n* = 2), optical coherence tomography (OCT) images (*n* = 2), cone‐beam computed tomography (CBCT) images (*n* = 2), and medical records/data sets (*n* = 2) (Figure 2).

The most often employed algorithms in the included research were the multilayer perceptron (*n* = 3), support vector machine (SVM) (*n* = 3), CNN models (*n* = 6), and ANN (*n* = 3). The algorithms were built to perform the following tasks: segmentation (*n* = 4), classification (*n* = 6), caries detection (*n* = 4), caries diagnosis (*n* = 4), and caries prediction (*n* = 2). Only two studies out of seven revealed the forms of caries that were frequently found, including pre‐cavitated lesions, initial caries, occlusal proximal, root, enamel, and dentinal lesions, primarily affecting premolar, or molar teeth(Prados‐Privado et al. 2020; Talpur et al. 2022).

### 3.5 Summary of the Performance Metrics of the Algorithms

Six studies examined the caries detection models' accuracy, which varied from 65.7% for RetinaNet to 99.1% for VGG Net‐16. Periapical radiographs and panoramic radiographs had the highest accuracy among the data sets, ranging from 82% to 99.2% and 86.1% to 96.1%, respectively, while near‐infrared transillumination images had the lowest accuracy, ranging from 68.0% to 78.0%. The sensitivity metrics of the AI modes were revealed in four studies. Deep learning models demonstrated the highest sensitivity in the caries detection, segmentation, and classification test, ranging from 25% to 99.7% (Mohammad‐Rahimi et al. 2022) while neural networks demonstrated a sensitivity of 59% to 99.6% (Khanagar et al. 2022). Five studies out of seven reported model specificity, with customized CNN models displaying the highest specificity (81% to 100%) (Moharrami et al. 2024). AUC, which assesses the overall efficacy of the binary classification mode, classifies negative results as AUC 0.7. In four investigations (Khanagar et al. 2022; Prados‐Privado et al. 2020), the AUC for neural networks was observed to range from 0.69 to 0.99. In contrast, machine learning models provide AUC for caries diagnosis ranging from 0.740 to 0.987; for proximal caries lesion detection from 0.74 to 0.917; for caries classification from 0.857 to 0.987; and segmentation from 0.836 for occlusal lesions to 0.856 for proximal lesions (Reyes et al. 2022). Only two studies (Khanagar et al. 2022; Prados‐Privado et al. 2020) examined the positive predictive value of neural networks, which ranged from 63% to 99%. In a similar vein, only two investigations(Khanagar et al. 2022; Moharrami et al. 2024) revealed negative predictive values, with values ranging from 73% to 98.15% (Table 3).

**Table 3:** AI performance indicators in included studies and significance/direction.

Author (year)	Year	AI performance indicators						Significance/direction
		Accuracy	Sensitivity	Specificity	Area under curve	PPV	NPV	
Prados‐Privado et al. (2020)	2020	68.57%–99%	NA	NA	0.74 to 0.971 (mean ± SD of 0.815 ± 0.1)	68.57%–99%	NR	Due to varied diagnostic performance, comparing algorithmic task results is essential before the clinical implementation of AI
Khanagar et al. (2022)	2022	69%–97.1%	59% to 99.6%	11%–98.19%	0.69 to 0.99	63%–93.48%	73% to 98.15%	These models increase diagnostic precision, but for improved performance, further training and validation are needed
Revilla‐León et al. (2022)	2022	76%–88.3%	73% to 90%	61.5%–93%	NR	NR	NR	By combining intraoral scans with image data to increase accuracy in restorative dentistry, AI can improve dental caries diagnosis
Mohammad‐Rahimi et al. (2022)	2022	Intra‐oral photographs: 71%–96%; peri‐apical radiographs: 82%–99.2%; bitewing radiographs: 87.6%–95.4%; NILT images: 68.0%–78.0%, on OCT images: 88.7%–95.2%, and panoramic radiographs: 86.1%–96.1%	25% to 99.8%	17.2%–100%	NR	NR	NR	Deep learning's significant caries detection (typically > 80%) has been highlighted in studies, emphasizing the necessity for reliable reference tests and clinically applicable results in future investigations
Talpur et al. (2022)	2022	CNN: 70%–93.30%; ANN: 69%; NN: 88.04%–99%; SVM; 92.37%–97%; ADA + NN: 95%	NR	NR	NR	NR	NR	With a stunning 99% accuracy in caries identification, backpropagation in neural networks is the best option for caries detection
Reyes et al. (2022)	2022	NR	0.000 to 0.971 (mean 0.669, SD 0.420)	0.679 and 0.993 (mean 0.866, SD 0.121)	Diagnosis: 0.740 to 0.987; proximal caries lesion detection: 0.74 to 0.917; caries classification: 0.857 to 0.987; segmentation: 0.836 and 0.856 for occlusal and proximal caries	NR	NR	Although studies offer compelling data, independent sample validation and cost‐effectiveness analyses are crucial for the adoption of practical technology
Moharrami et al. (2024)	2023	RetinaNet: 65.7% to VGG NET‐16: 99.1%	NR	81.5%–100.0% (customized CNN)	NR	NR	77.3%–96.2%	Due to a lack of research on varied lesion severity, AI's role in caries diagnosis is critical. Additional experimentation with smartphone‐derived images is required

Abbreviations: ANN, artificial neural network; CNN, convolutional neural networks; NN, neural network; NPV, negative predictive value; NR, not reported; PPV, positive predictive value; SVM, support vector machine.

### 3.6 Quality Appraisal Findings

**Figure 3:**
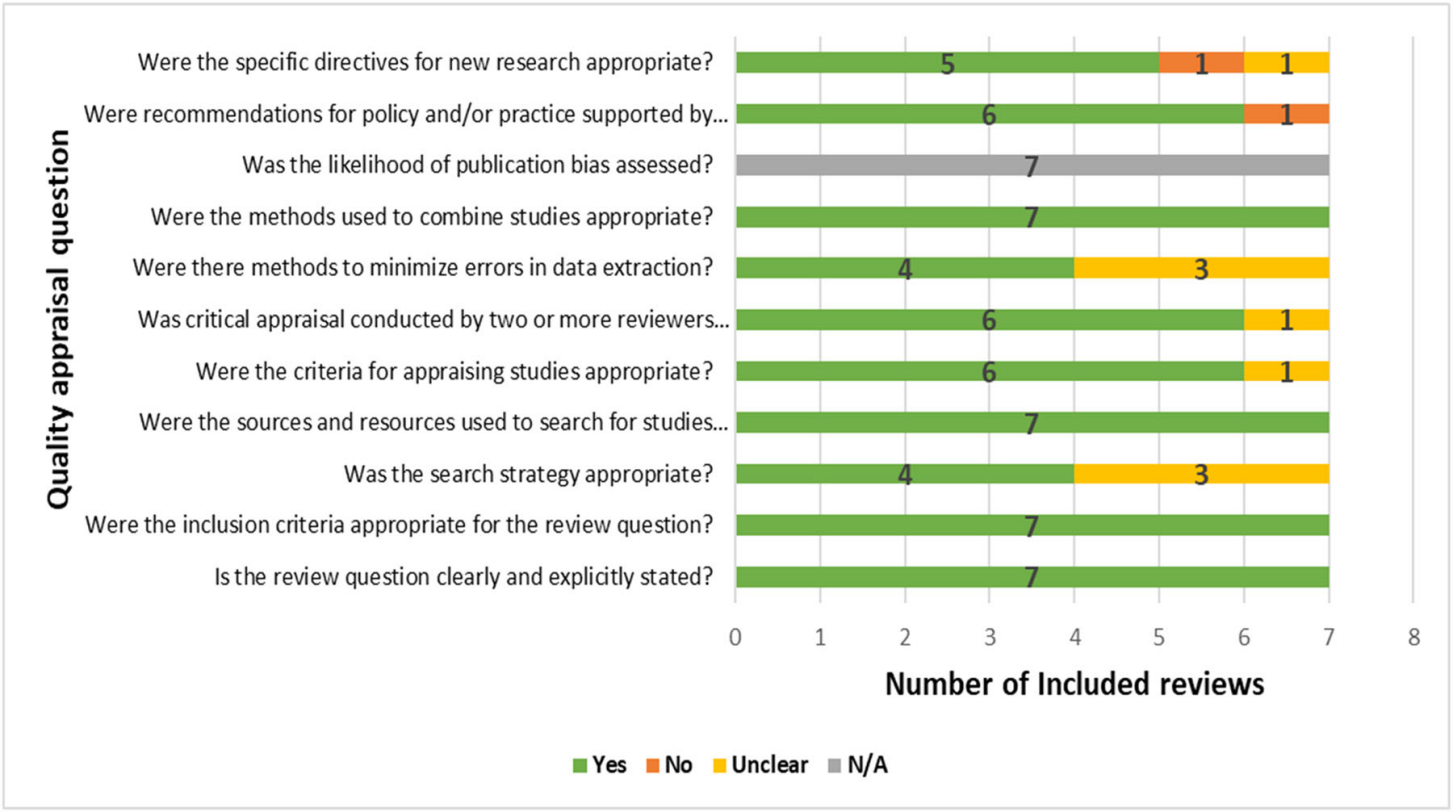
Quality appraisal of included reviews.

All included reviews consistently and precisely described their review questions, inclusion criteria, sources of search, and appropriately synthesized data. However, only four studies showed a clear and comprehensive search strategy that included all pertinent search terms, subject headings, and restrictions. Except for one study (Talpur et al. 2022), which did not specify the instrument used for quality appraisal, quality assessments of the included studies were consistently completed, with the standard tool utilized by two or more reviewers independently, while Talpur et al. (2022) review did not adhere to this standard procedure. Four reviews included at least two experts who separately extracted data using a structured data extraction sheet to reduce bias and errors in data extraction. However, as no review included a meta‐analysis, none of the reviews evaluated the likelihood of publication bias. Except for Revilla‐León et al. (2022), all investigations offered pertinent, practical implications for their conclusions. All studies presented pertinent future research implications based on their findings, apart from Khanagar et al. (2022) and Mohammad‐Rahimi et al. (2022). Overall, there was a low risk of bias in the included reviews (Figure 3).

## 4 Discussion

This umbrella review included seven systematic reviews to summarize the application and effectiveness of AI models in dental caries detection and diagnosis. A protocol's development and registration are critical components of a high‐quality systematic review because they provide a clear roadmap for the study's objectives, methods, and intended analysis. Unfortunately, five of the studies considered in this study did not have their protocols registered in any public repository, which raises issues regarding potential bias and selective reporting. A registered protocol not only promotes transparency but also safeguards against the possibility of outcome reporting bias, unanticipated work repetition, and resource waste, ultimately enhancing scientific standards (Johnston et al. 2019; Stewart, Moher, and Shekelle 2012). To further guarantee the caliber and thoroughness of systematic reviews, adherence to well‐established reporting protocols, such as PRISMA and PRISMA‐DTA, is essential (Gagnier and Kellam 2013). Accurate judgments and the correct interpretation of the findings and conclusions might be hampered by inadequate reporting, which can cloud the review's conduct (Frank, Bossuyt, and McInnes 2018; Moher, Stewart, and Shekelle 2016). To increase the accuracy and openness of their systematic reviews and eventually benefit the larger scientific community, researchers must insist on protocol registration and standard guideline compliance.

The PICO model was created to aid in the structure of a well‐constructed research question to facilitate a literature search. It served a significant role as a conceptualizing structure for evidence‐based healthcare (Eriksen and Frandsen 2018). The difference in PICO structure identified throughout the included systematic studies highlights the complexity and diversity of AI applications in dental caries. One noticeable distinction is the lack of clarity in the target population characteristics in some studies. For example, Revilla‐León et al. (2022) defined the population as clinical applications in restorative dentistry for the identification of dental caries, whereas Prados‐Privado et al. (2020) defined the population as neural networks and dental caries. This ambiguity might have an impact on the precision and usefulness of the results, underscoring the significance of accurately defining the population under study. In terms of comparators, expert judgment, clinical examination, or reference tests were the most commonly used, mirroring real‐world clinical circumstances. However, the potential shortcomings and subjectivity associated with human judgments must be acknowledged. Talpur et al. (2022) provided an alternate strategy where machine learning approaches were used as comparators, offering a more objective basis for comparison, which can help alleviate the bias inherent in expert judgment. Furthermore, the reported result indicators varied greatly. Moharrami et al. (2024), for example, used the F1 score‐ a performance matric that combinedly indicates precision and recall of classifier (Alakus and Turkoglu 2020) while Khanagar et al. (2022) used various metrics. Because of the heterogeneity in performance standards, we could not perform meta‐analyses and make conclusive findings. Thus, future research should develop shared frameworks and standardized measurements to allow for clearer comparisons across studies and contribute to a more thorough knowledge of the topic.

In terms of key findings, CNNs—a class of deep neural networks used for image classification and computer vision tasks, consistently demonstrated high levels of specificity, sensitivity, and AUC values, all of which are critical for accurate detection, diagnosis, and prediction of dental caries. Notably, Talpur et al. (2022) found that the neural network backpropagation algorithm (an algorithm to train artificial neural networks) was the best choice for dental image data sets, obtaining a remarkable accuracy rate of up to 99%. In the research done by Dayı et al. (2023) different CNN‐based algorithms, such as MobileNetV2, VGG16, ResNet50, EfficientNet, and Inception network architectures, were assessed for their ability to detect occlusal, proximal, and cervical caries. The results of the study demonstrated the effectiveness of AI systems based on deep learning, particularly the ResNet50‐DCDNet network, which correctly identified occlusal and proximal caries and received the highest F1 score of 62.7%. These findings highlight how deep learning‐based methods and CNNs have the potential to dramatically improve the precision and effectiveness of dental caries identification, opening interesting new directions for dental diagnostics and patient care. Zhu et al. (2022) tested CariesNet, a CNN‐based architectural innovation, to interpret dental radiographs using AI and machine learning. In comparison to conventional manual inspection, it obtains a remarkable accuracy rate of 93.61% in detecting the presence of dental caries. These findings highlight CNNs' tremendous potential for improving the precision and efficiency of caries identification and diagnosis in dental practice. This development has the potential to improve not only patient outcomes but also healthcare processes. Furthermore, as shown by Schwendicke et al. (2021), the use of AI for caries diagnosis is expected to be cost‐effective, particularly in terms of reducing undiscovered lesions.

A critical key finding from the study reveals the greater accuracy of periapical and panoramic radiography in detecting dental caries. It matches with the conclusions reached by Turosz et al. (2023) in their umbrella review of AI applications in panoramic radiograph analysis. Their review emphasized that AI applications can significantly assist dentists in analyzing dental panoramic radiographs, especially in dental caries, with an incredible precision rate of 91.5%. Furthermore, Oztekin et al. (2023) used panoramic radiography pictures to test three deep learning‐driven models with prior training for automated dental caries detection. Among these models, ResNet‐50, an image classification model consisting of 50 CNN layers, performed best, with an accuracy of 92.00%, sensitivity of 87.33%, and F1‐score of 91.61%, demonstrating its efficacy in caries detection. However, ethical and mindful adoption is needed for the incorporation of AI in dentistry (Anil, Porwal, and Porwal 2023).

In addition, the widespread availability of high‐quality AI solutions in dentistry could culminate in the emergence of a new professional area centered on AI‐driven detection and treatments (Karobari et al. 2023). However, the emphasis should go beyond sample sizes to emphasize its generalizability and reproducibility. Moreover, transparency must be ensured by researchers by sharing both data and algorithm codes, allowing for rigorous validation, and increasing trust in AI‐driven dentistry diagnoses and therapies. Despite these prospects and strengths of AI in dental caries diagnosis and detection, considerable problems remain in sharing data and management. Personal patient data are required for the training, validation, and enhancement of AI algorithms, which necessitates data sharing between institutions or beyond national boundaries. Data security is therefore essential for the successful integration of AI into clinical practice. Mechanisms are required to guarantee the accuracy of AI algorithms and resolve the question of who is responsible for errors made by the technology. The transition from human to autonomous agents presents substantial ethical and legal issues, raising doubts about our legal system's capacity to adjust to the changing role of AI. Besides, data quality and AI algorithm transparency are essential since inadequate labeling can hinder AI's effectiveness in dentistry. For AI to make clear healthcare decisions, interpretability must be improved through research (Joda et al. 2019).

### 4.1 Limitations and Future Recommendations

The fundamental drawback of this study derives from the wide variance in AI algorithms, data sources, and performance measures observed among the included experiments. CNNs, ANNs, RetinaNet, VGG models, and other techniques were used to analyze data from several sources, including periapical radiographs, smartphone images, bitewing radiographs, and NILT images. Furthermore, classifier effectiveness was evaluated using various measures, such as accuracy, sensitivity, specificity, ROC analysis, PPV, and NPV. Owing to this wider heterogeneity, statistical analysis of the data was impossible for this umbrella review. The presence of primary studies that may have been duplicated throughout the included reviews is another key limitation of this umbrella review. Because of this overlap in original research across included systematic reviews, published classifier performance ranges may be misleading. Moreover, our umbrella review relies on existing systematic reviews and meta‐analyses, so there is a risk of potentially excluding the latest evidence from studies not yet included in such analyses (Gianfredi et al. 2022).

Future umbrella reviews must include a thorough screening strategy to uncover and reject duplicate studies, ensuring that the results are based on distinct data sets and findings. Moreover, Future research should focus on ensuring consistency in AI algorithms, data formats, and evaluation metrics to facilitate direct comparison Besides this, there is a great deal of potential for using smartphones to take dental imaging pictures. However, this has rarely been studied by researchers so far. Future studies must test the incorporation of AI technologies in the identification of dental caries using these devices to extract crucial factors from dental photos, thus, increasing the availability of dental health evaluations and enhancing the precision and application of AI‐based caries detection technologies. Furthermore, to assess the real‐time clinical implication of AI models, longitudinal studies in real‐world settings should be carried out. An interdisciplinary collaboration between experts from dentistry, computer science, and radiology as well as regulatory bodies to meet end‐users' demands along with safety, accuracy, and reliability standards is crucial for advancing the ethical integration of AI in dental practice.

## 5 Conclusion

AI models, especially CNN‐based models, have an enormous amount of potential for accurate, objective dental caries diagnosis and detection. This emphasizes the promising role of AI in improving the precision and effectiveness of caries detection and diagnosis in dentistry practice, with the potential to enhance patient outcomes and streamline healthcare procedures. However, ensuring end‐users' demands along with safety, accuracy, and reliability standards remain critical to its successful integration into routine practice.

## Author Contributions

All authors have equal contributions to the study's conception and design. Material preparation, data curation, and analysis were performed by S.N. and A.M. S.N. wrote the first draft of the manuscript and all authors (A.M., S.T., N.T.S.S., A.H.A., M.I.K., and V.M.) commented on previous versions. All authors read and approved the final manuscript.

## Ethics Statement

The authors have nothing to report.

## Conflicts of Interest

The authors declare no conflicts of interest.

## Supporting information

Supporting information.

## Data Availability

Data sharing does not apply to this article as no new data were created or analyzed in this study.

## References

[cre270004-bib-0001] Ahmed, W. M. , A. A. Azhari , K. A. Fawaz , et al. 2023. “Artificial Intelligence in the Detection and Classification of Dental Caries.” *Journal of Prosthetic Dentistry*. Published ahead of print, August 26, 2023. 10.1016/j.prosdent.2023.07.013. 37640607

[cre270004-bib-0002] Alakus, T. B. , and I. Turkoglu . 2020. “Comparison of Deep Learning Approaches to Predict COVID‐19 Infection.” Chaos, Solitons, and Fractals 140: 110120. 10.1016/j.chaos.2020.110120. 33519109 PMC7833512

[cre270004-bib-0003] Anil, S. , P. Porwal , and A. Porwal . 2023. “Transforming Dental Caries Diagnosis Through Artificial Intelligence‐Based Techniques.” Cureus 15, no. 7: e41694. 10.7759/cureus.41694. 37575741 PMC10413921

[cre270004-bib-0004] Dayı, B. , H. Üzen , İ. B. Çiçek , and Ş. B. Duman . 2023. “A Novel Deep Learning‐Based Approach for Segmentation of Different Type Caries Lesions on Panoramic Radiographs.” Diagnostics 13, no. 2: Article 2. 10.3390/diagnostics13020202. PMC9858411 36673010

[cre270004-bib-0005] Eriksen, M. B. , and T. F. Frandsen . 2018. “The Impact of Patient, Intervention, Comparison, Outcome (PICO) as a Search Strategy Tool on Literature Search Quality: A Systematic Review.” Journal of the Medical Library Association 106, no. 4: 420–431. 10.5195/jmla.2018.345. 30271283 PMC6148624

[cre270004-bib-0006] Frank, R. A. , P. M. Bossuyt , and M. D. F. McInnes . 2018. “Systematic Reviews and Meta‐Analyses of Diagnostic Test Accuracy: The PRISMA‐DTA Statement.” Radiology 289, no. 2: 313–314. 10.1148/radiol.2018180850. 30015590

[cre270004-bib-0007] Gagnier, J. J. , and P. J. Kellam . 2013. “Reporting and Methodological Quality of Systematic Reviews in the Orthopaedic Literature.” Journal of Bone and Joint Surgery 95, no. 11: e77. 10.2106/JBJS.L.00597. 23780547

[cre270004-bib-0008] Gianfredi, V. , D. Nucci , A. Amerio , C. Signorelli , A. Odone , and M. Dinu . 2022. “What Can We Expect From an Umbrella Review?.” Advances in Nutrition 13, no. 2: 684–685. 10.1093/advances/nmab150. 37270205 PMC8970837

[cre270004-bib-0009] Gimenez, T. , C. Piovesan , M. M. Braga , et al. 2015. “Visual Inspection for Caries Detection: A Systematic Review and Meta‐Analysis.” Journal of Dental Research 94, no. 7: 895–904. 10.1177/0022034515586763. 25994176

[cre270004-bib-0010] Jain, N. , U. Dutt , I. Radenkov , and S. Jain . 2024. “WHO's Global Oral Health Status Report 2022: Actions, Discussion and Implementation.” Oral Diseases 30, no. 2: 73–79. 10.1111/odi.14516. 36680388

[cre270004-bib-0011] JBI . n.d. “Critical Appraisal Tools.” Accessed January 1, 2023. https://jbi.global/critical-appraisal-tools.

[cre270004-bib-0012] JBI . 2020. “Chapter 10: Umbrella Reviews.” In *JBI Manual for Evidence Synthesis*, edited by E. Aromataris and Z. Munn, 363–400. Adelaide, Australia: JBI. 10.46658/JBIMES-20-11.

[cre270004-bib-0013] Joda, T. , T. Waltimo , N. Probst‐Hensch , C. Pauli‐Magnus , and N. U. Zitzmann . 2019. “Health Data in Dentistry: An Attempt to Master the Digital Challenge.” Public Health Genomics 22, no. 1/2: 1–7. https://www.jstor.org/stable/26788803. 31390644 10.1159/000501643

[cre270004-bib-0014] Johnston, A. , S. E. Kelly , S.‐C. Hsieh , B. Skidmore , and G. A. Wells . 2019. “Systematic Reviews of Clinical Practice Guidelines: A Methodological Guide.” Journal of Clinical Epidemiology 108: 64–76. 10.1016/j.jclinepi.2018.11.030. 30529647

[cre270004-bib-0015] Karobari, M. I. , A. H. Adil , S. N. Basheer , et al. 2023. “Evaluation of the Diagnostic and Prognostic Accuracy of Artificial Intelligence in Endodontic Dentistry: A Comprehensive Review of Literature.” Computational and Mathematical Methods in Medicine 2023: e7049360. 10.1155/2023/7049360. PMC9904932 36761829

[cre270004-bib-0016] Kazeminia, M. , A. Abdi , S. Shohaimi , et al. 2020. “Dental Caries in Primary and Permanent Teeth in Children's Worldwide, 1995 to 2019: A Systematic Review and Meta‐Analysis.” Head & Face Medicine 16, no. 1: 22. 10.1186/s13005-020-00237-z. 33023617 PMC7541284

[cre270004-bib-0017] Khanagar, S. B. , K. Alfouzan , M. Awawdeh , L. Alkadi , F. Albalawi , and A. Alfadley . 2022. “Application and Performance of Artificial Intelligence Technology in Detection, Diagnosis and Prediction of Dental Caries (DC)—A Systematic Review.” Diagnostics 12, no. 5: Article 5. 10.3390/diagnostics12051083. PMC9139989 35626239

[cre270004-bib-0018] Mertens, S. , J. Krois , A. G. Cantu , L. T. Arsiwala , and F. Schwendicke . 2021. “Artificial Intelligence for Caries Detection: Randomized Trial.” Journal of Dentistry 115: 103849. 10.1016/j.jdent.2021.103849. 34656656

[cre270004-bib-0019] Mohammad‐Rahimi, H. , S. R. Motamedian , M. H. Rohban , et al. 2022. “Deep Learning for Caries Detection: A Systematic Review.” Journal of Dentistry 122: 104115. 10.1016/j.jdent.2022.104115. 35367318

[cre270004-bib-0020] Moharrami, M. , J. Farmer , S. Singhal , et al. 2024. “Detecting Dental Caries on Oral Photographs Using Artificial Intelligence: A Systematic Review.” Oral Diseases 30: 1765–1783. 10.1111/odi.14659. 37392423

[cre270004-bib-0021] Moher, D. , L. Stewart , and P. Shekelle . 2016. “Implementing PRISMA‐P: Recommendations for Prospective Authors.” Systematic Reviews 5, no. 1: 15. 10.1186/s13643-016-0191-y. 26822481 PMC4730599

[cre270004-bib-0022] Muñoz‐Sandoval, C. , K. Gambetta‐Tessini , J. N. Botelho , and R. A. Giacaman . 2022. “Detection of Cavitated Proximal Carious Lesions in Permanent Teeth: A Visual and Radiographic Assessment.” Caries Research 56, no. 3: 171–178. 10.1159/000525193. 35605576

[cre270004-bib-0023] Oztekin, F. , O. Katar , F. Sadak , et al. 2023. “An Explainable Deep Learning Model to Prediction Dental Caries Using Panoramic Radiograph Images.” Diagnostics 13, no. 2: Article 2. 10.3390/diagnostics13020226. PMC9858273 36673036

[cre270004-bib-0024] Page, M. J. , J. E. McKenzie , P. M. Bossuyt , et al. 2021. “The PRISMA 2020 Statement: An Updated Guideline for Reporting Systematic Reviews.” BMJ 372: n71. 10.1136/bmj.n71. 33782057 PMC8005924

[cre270004-bib-0025] Patil, S. , S. Albogami , J. Hosmani , et al. 2022. “Artificial Intelligence in the Diagnosis of Oral Diseases: Applications and Pitfalls.” Diagnostics 12, no. 5: 1029. 10.3390/diagnostics12051029. 35626185 PMC9139975

[cre270004-bib-0026] Prados‐Privado, M. , J. García Villalón , C. H. Martínez‐Martínez , C. Ivorra , and J. C. Prados‐Frutos . 2020. “Dental Caries Diagnosis and Detection Using Neural Networks: A Systematic Review.” Journal of Clinical Medicine 9, no. 11: Article 11. 10.3390/jcm9113579. PMC7694692 33172056

[cre270004-bib-0027] PROSPERO International Prospective Register of Systematic Reviews . 2023. “Application and Effectiveness of Artificial Intelligence in Dental Caries Diagnosis and Management: An Umbrella Review.” https://www.crd.york.ac.uk/prospero/display_record.php?RecordID=464376.

[cre270004-bib-0028] Ramos‐Gomez, F. , M. Marcus , C. A. Maida , et al. 2021. “Using a Machine Learning Algorithm to Predict the Likelihood of Presence of Dental Caries Among Children Aged 2 to 7.” Dentistry Journal 9, no. 12: Article 12. 10.3390/dj9120141. PMC8700143 34940038

[cre270004-bib-0029] Revilla‐León, M. , M. Gómez‐Polo , S. Vyas , et al. 2022. “Artificial Intelligence Applications in Restorative Dentistry: A Systematic Review.” Journal of Prosthetic Dentistry 128, no. 5: 867–875. 10.1016/j.prosdent.2021.02.010. 33840515

[cre270004-bib-0030] Reyes, L. T. , J. K. Knorst , F. R. Ortiz , and T. M. Ardenghi . 2022. “Machine Learning in the Diagnosis and Prognostic Prediction of Dental Caries: A Systematic Review.” Caries Research 56, no. 3: 161–170. 10.1159/000524167. 35636386

[cre270004-bib-0031] Schwendicke, F. , J. G. Rossi , G. Göstemeyer , et al. 2021. “Cost‐Effectiveness of Artificial Intelligence for Proximal Caries Detection.” Journal of Dental Research 100, no. 4: 369–376. 10.1177/0022034520972335. 33198554 PMC7985854

[cre270004-bib-0032] Stewart, L. , D. Moher , and P. Shekelle . 2012. “Why Prospective Registration of Systematic Reviews Makes Sense.” Systematic Reviews 1, no. 1: 7. 10.1186/2046-4053-1-7. 22588008 PMC3369816

[cre270004-bib-0033] Talpur, S. , F. Azim , M. Rashid , S. A. Syed , B. A. Talpur , and S. J. Khan . 2022. “Uses of Different Machine Learning Algorithms for Diagnosis of Dental Caries.” Journal of Healthcare Engineering 2022: e5032435. 10.1155/2022/5032435. PMC8989613 35399834

[cre270004-bib-0034] Tripathy, S. , A. Mathur , and V. Mehta . 2023. “A View of Neural Networks in Artificial Intelligence in Oral Pathology.” Oral Surgery 17, no. 2: 179–180. 10.1111/ors.12848.

[cre270004-bib-0035] Turosz, N. , K. Chęcińska , M. Chęciński , A. Brzozowska , Z. Nowak , and M. Sikora . 2023. “Applications of Artificial Intelligence in the Analysis of Dental Panoramic Radiographs: An Overview of Systematic Reviews.” Dentomaxillofacial Radiology 52, no. 7: 20230284. 10.1259/dmfr.20230284. 37665008 PMC10552133

[cre270004-bib-0036] WHO . 2022. Oral Health. Geneva, Switzerland: World Health Organization. https://www.who.int/news-room/fact-sheets/detail/oral-health.

[cre270004-bib-0037] Zhu, Y. , T. Xu , L. Peng , et al. 2022. “Faster‐RCNN Based Intelligent Detection and Localization of Dental Caries.” Displays 74: 102201. 10.1016/j.displa.2022.102201.

