# Supplementary material for: Artificial Intelligence in Dental Caries Diagnosis and Detection: An Umbrella Review
Source: Clin Exp Dent Res. 2024 Aug 29;10(4):e70004. doi: 10.1002/cre2.70004 (PMC11358700; doi:10.1002/cre2.70004)
Supplement: Supplementary file 1 — Supporting information. [file CRE2-10-e70004-s001.docx]

**Supplementary Table 1: Search strategy**

| **Database** | **Search Terms** | **Results** |
| --- | --- | --- |
| PubMed | ("Artificial Intelligence"[Mesh] OR "Machine Learning"[Mesh] OR "Neural Networks, Computer"[Mesh] OR Expert Systems [Mesh] OR (AI)) AND ("Dental Caries"[Mesh] OR tooth decay OR caries) | [576](https://pubmed.ncbi.nlm.nih.gov/?term=%28%22Artificial+Intelligence%22%5BMesh%5D+OR+%22Machine+Learning%22%5BMesh%5D+OR+%22Neural+Networks%2C+Computer%22%5BMesh%5D+OR+Expert+Systems%5BMesh%5D+OR+%28AI%29%29+AND+%28%22Dental+Caries%22%5BMesh%5D+OR+tooth+decay+OR+caries%29&sort=relevance) |
| Cochrane Library | "artificial intelligence" OR AI OR "Machine Learning" OR "neural network" OR expert system in All Text AND "dental caries" OR oral caries OR tooth decay in All Text AND "systematic review" OR meta-analysis in All Text | 83 |
| IEEE Explore | ("All Metadata":Artificial Intelligence OR "All Metadata":AI or Machine learning OR "All Metadata":neural networks OR "All Metadata":expert system) AND ("All Metadata":Dental Caries OR "All Metadata":Oral Caries OR "All Metadata":tooth decay OR "All Metadata":caries) | 70 |
| Embase | 'artificial intelligence'/exp OR 'artificial intelligence' OR ai OR 'machine learning'/exp OR 'machine learning' OR 'artificial neural network'/exp OR 'artificial neural network' OR 'expert system'/exp OR 'expert system'  system'/exp OR 'expert system' AND dentistry' OR 'dental caries' AND 'review'/it | 520 |
|  | **Total studies found** | **1249** |

**Supplementary Table 2:** **Study search, appraisal and synthesis**

| **Author/Year** | **Database** | **Reference list checking** | **Critical appraisal tool** | **Critical appraisal findings** | **Data synthesis** |
| --- | --- | --- | --- | --- | --- |
| Prados-Privado et al. 2020 | MEDLINE/PubMed, Institute of Electrical and Electronics Engineers (IEEE) Xplore, and ScienceDirect | Yes | Cochrane risk of bias assessment tool | in most domains, no data were given related to the transparency of the studies. | mean, standard deviation (SD), median, and percentage |
| Khanagar et al. 2022 | PubMed, Google scholar, Scopus, Web of science, Embase, Cochrane, Saudi Digital Library) | Yes | QUADAS-2 | majority of studies have low risk and a very small number of studies show high risk of bias. | Narrative |
| Revilla et al 2022 | MEDLINE/PubMed, EMBASE, World of Science, Cochrane, and Scopus | NR | Joanna Briggs Institute (JBI) Critical Appraisal Checklist for Quasi-Experimental Studies | 100% low risk of bias in all included articles for questions 1, 8, and 9. For question 4, 60% of low risk and 40% of high risk of bias was computed. questions 2 and 6 of the JBI were not applicable in this systematic review. Questions 3, 5, and 7 were not applicable | Narrative |
| Mohammad et al. 2022 | Medline (via PubMed), Google Scholar, Scopus, Embase, and ArXiv | Yes | QUADAS-2 tool | Among the included studies, 11 (26.2%) were found to have low risk of biases in all four domains. Moreover, 13 studies (31.0%) were evaluated as low risk for concerns regarding applicability. The most problematic domain was “Reference Standard”, where only 20 studies (47.6%) and 22 studies (52.4%) were classified as low risk of bias and low risk of applicability concern, respectively. | Narrative |
| Talpur et al. 2022 | PubMed, IEEE Xplore, Science Direct, and Google scholar | NR | NR | 9 studies had low risk of bias and 3 had moderate risk of Bias | Descriptive |
| Reyes et al. 2022 | PubMed/Medline, Scopus, EMBASE, and Web of Science | Yes | QUADAS-2 | the majority of the diagnosis studies surveyed had a high or unclear bias risk across several domains of assessment, raising concerns about their applicability. | narrative synthesis |
| Moharrami et al. 2023 | Medline, Scopus, and EMBASE | Yes | QUADAS-2 | six studies were considered to have a low risk for all four domains of QUADAS-2. Regarding applicability concerns, seven studies were categorized as having low concern. | narrative synthesis |

QUADAS: Quality assessment of diagnostic Accuracy Studies; NR: Not reported

**Supplementary Table 3: Search results and dataset features in included studies**

| **Author/Year** | **No. of studies retrieved** | **No. of studies included** | **Data type** | **total image database, Oral photographs** |
| --- | --- | --- | --- | --- |
| **Prados-Privado et al. 2020** | 187 | 12 | periapical, the near-infrared light transilluminations, and the bitewings, panoramic radiographs, radiovisiography, intra-oral, in vivo with an intraoral camera and, and X-ray images | 87 to 3000 images, with a mean of 669.27 images, a standard deviation of 1153.76, and a median of 160 images |
| Khanagar et al. 2022 | 448 | 34 | periapical radiographs, Bitewing radiograph, digital radiographs, infrared transillumination (TI) images, near-infrared-light transillumination (NILT) images, smartphone photographs, Panoramic radiographs, OCT and micro-CT images, Oral photographs, datasets | not mentioned |
| Revilla et al 2022 | 1596 | 34 | periapical and/or bitewings radiographs, intraoral photographs, infrared transillumination techniques, fibre optic displacement sensor, CBCT images | NR |
| Mohammad et al. 2022 | 252 | 42 | intra-oral photographs, peri-apical radiographs, bitewing radiographs, optical coherence tomography images, panoramic radiographs, near-infrared light transillumination images, and cone-beam computed tomography | 80-7200 |
| Talpur et al. 2022 | 133 | 12 | dental radiographs | Training data: 80 to 9630 and testing data: from 80 to 2380 |
| Reyes et al. 2022 | 491 | 15 | radiographic, photographic, or near-infrared light transillumination [NILT] images bitwing radiographs, and medical records). | Training dataset: 80-3293; test dataset: 32-1818 |
| Moharrami et al. 2023 | 3410 | 19 | Professional camera, intraoral camera or smartphone images | 45 to 12,600 |
